# Supplementary material for: Geminiviruses employ host DNA glycosylases to subvert DNA methylation-mediated defense
Source: Nat Commun. 2022 Jan 31;13:575. doi: 10.1038/s41467-022-28262-3 (PMC8803994; doi:10.1038/s41467-022-28262-3)
Supplement: Supplementary file 1 — Supplementary Information [file 41467_2022_28262_MOESM1_ESM.pdf]

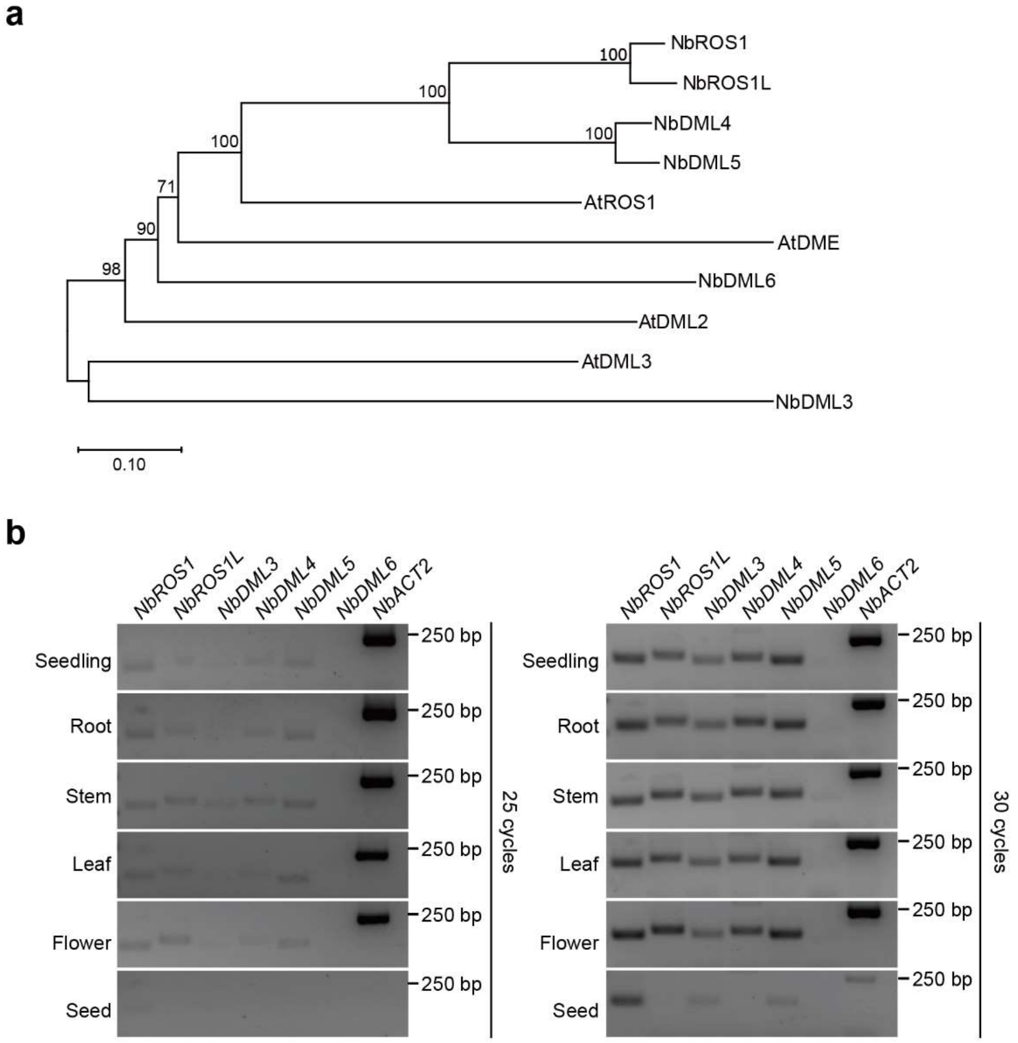

Supplementary Fig. 1 Characterization of *NbDML* genes. a Phylogenetic analysis of the DME family of DNA glycosylases in *N. benthamiana.* The phylogenetic tree was computed using the maximum likelihood method in MEGA v.7 based on the JTT model. The numbers at each node represent bootstrap values (n=1000). **b** Transcript levels of *NbROS1*, *NbROS1L*, *NbDML3*, *NbDML4*, *NbDML5*, and *NbDML6* in different tissues of *N. benthamiana*. Images in **b** are representative of three independent experiments. Uncropped gels for **b** are provided in the source data.

a

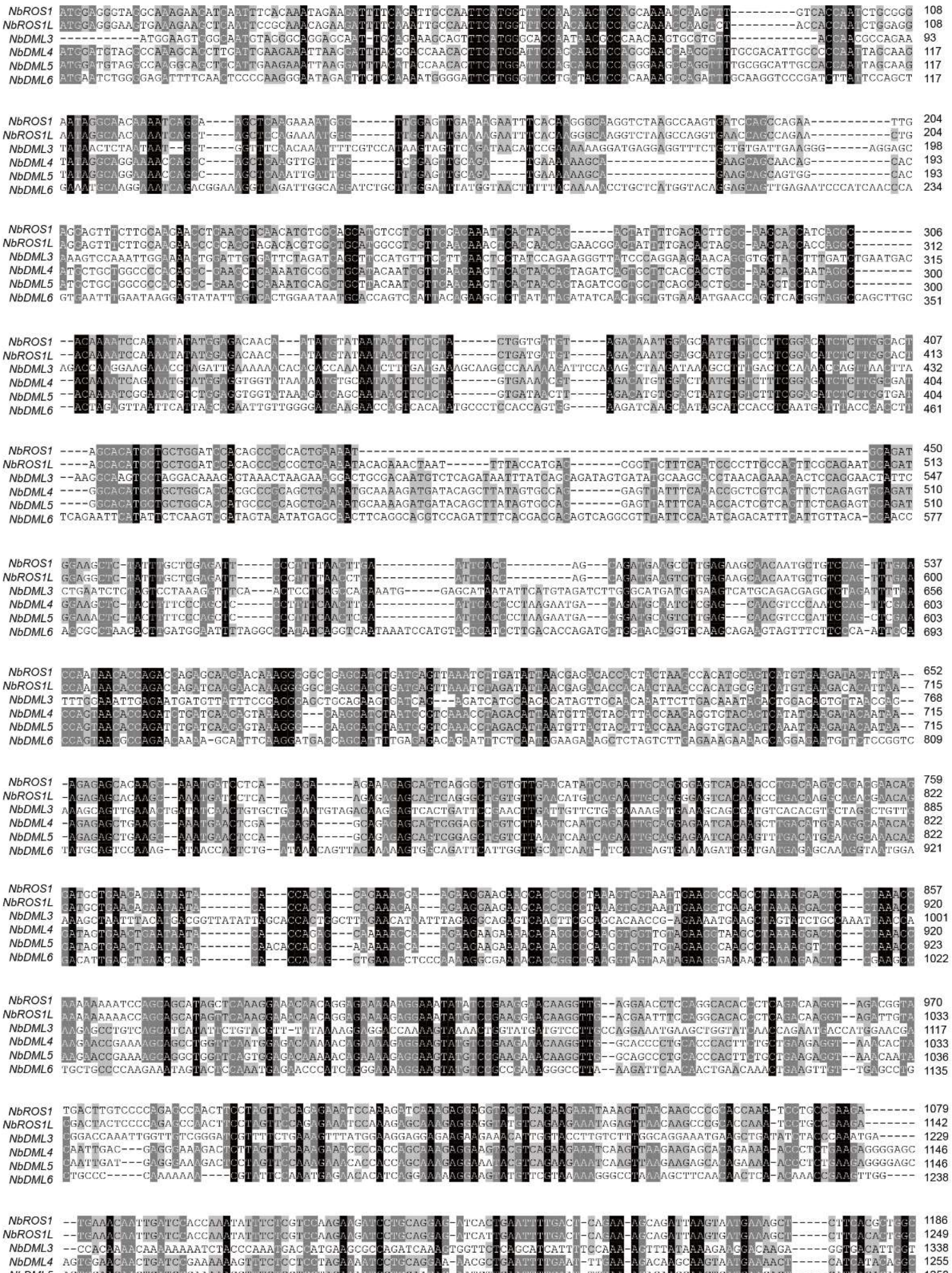
NbROS1
NbROS1L
NbDML3
NbDML4
NbDML5
NbDML6
108
108
93
117
117
117
204
204
198
193
193
234
306
312
315
300
300
351
407
413
432
404
404
461
450
513
547
510
510
577
537
600
656
603
603
693
652
715
768
715
715
809
759
822
885
822
822
921
857
920
1001
920
923
1022
970
1033
1117
1033
1036
1135
1079
1142
1229
1146
1146
1238
1186
1249
1338
1255
1258
1352

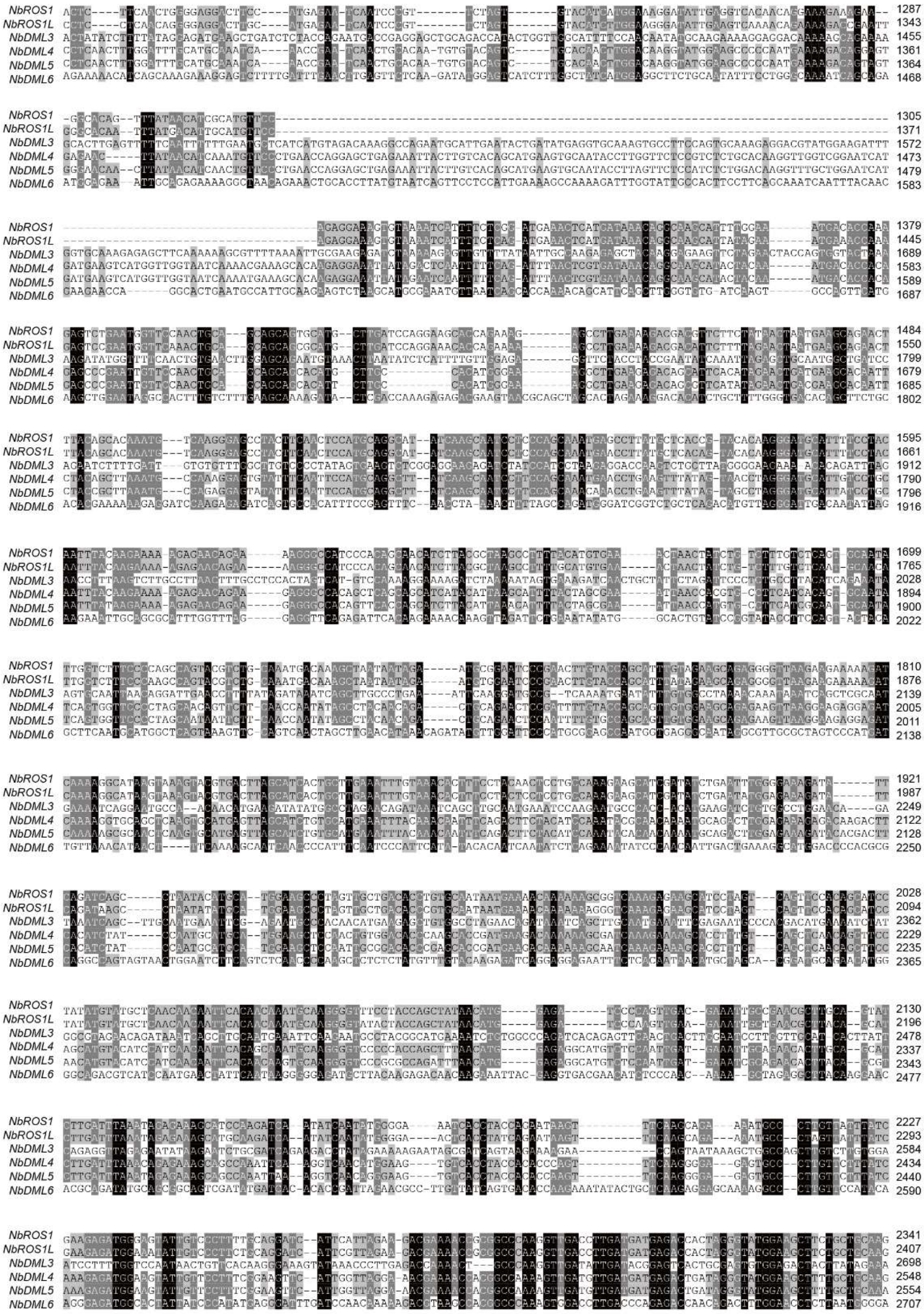

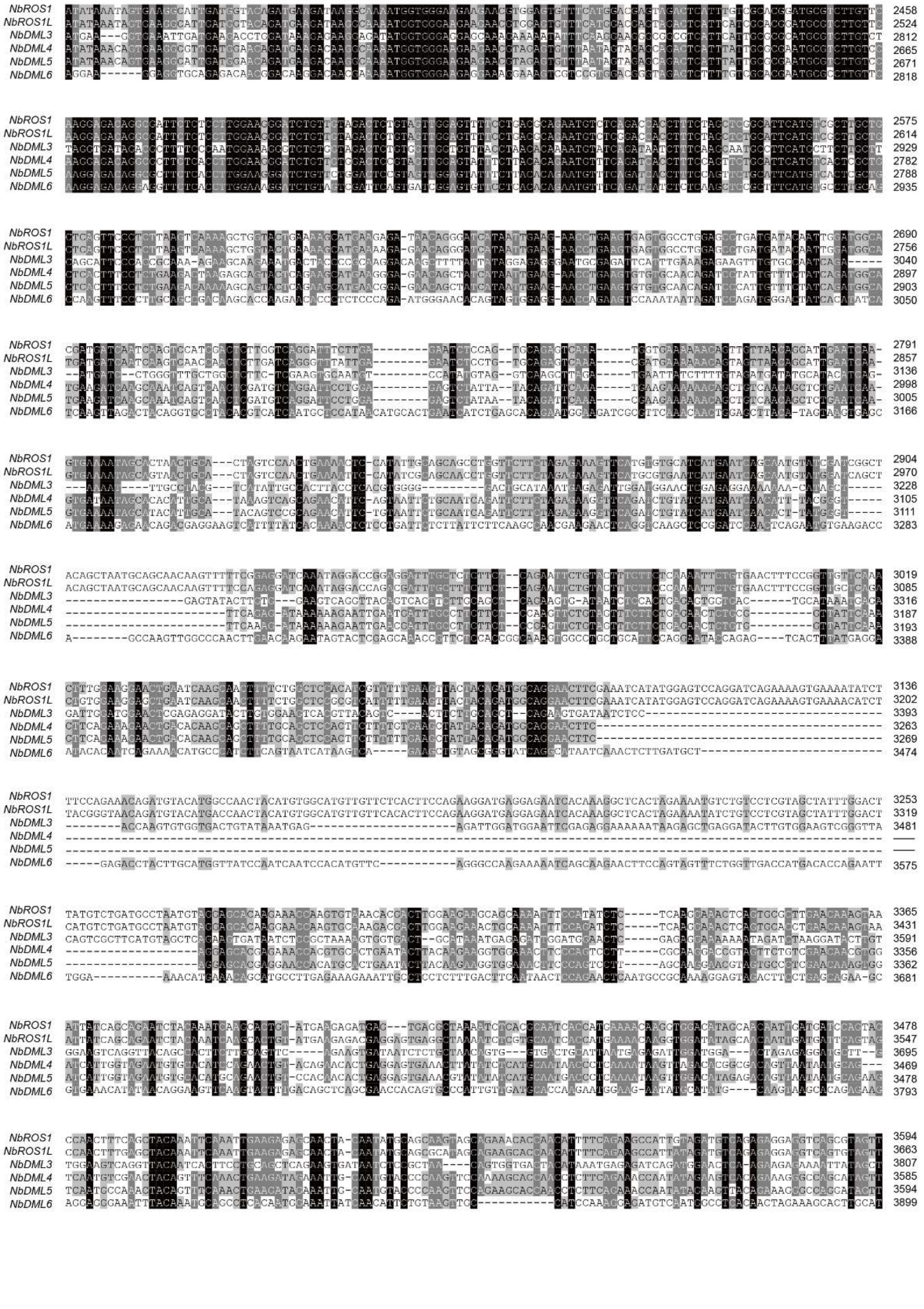
NbROS1
NbROS1L
NbDML3
NbDML4
NbDML5
NbDML6
2458
2524
2812
2665
2671
2818
2575
2614
2929
2782
2788
2935
2690
2756
3040
2897
2903
3050
2791
2857
3136
2998
3005
3166
2904
2970
3228
3105
3111
3283
3019
3085
3316
3187
3193
3388
3136
3202
3393
3263
3269
3474
3253
3319
3481
3575
3365
3431
3591
3356
3362
3681
3478
3547
3695
3469
3478
3793
3594
3663
3807
3585
3594
3899

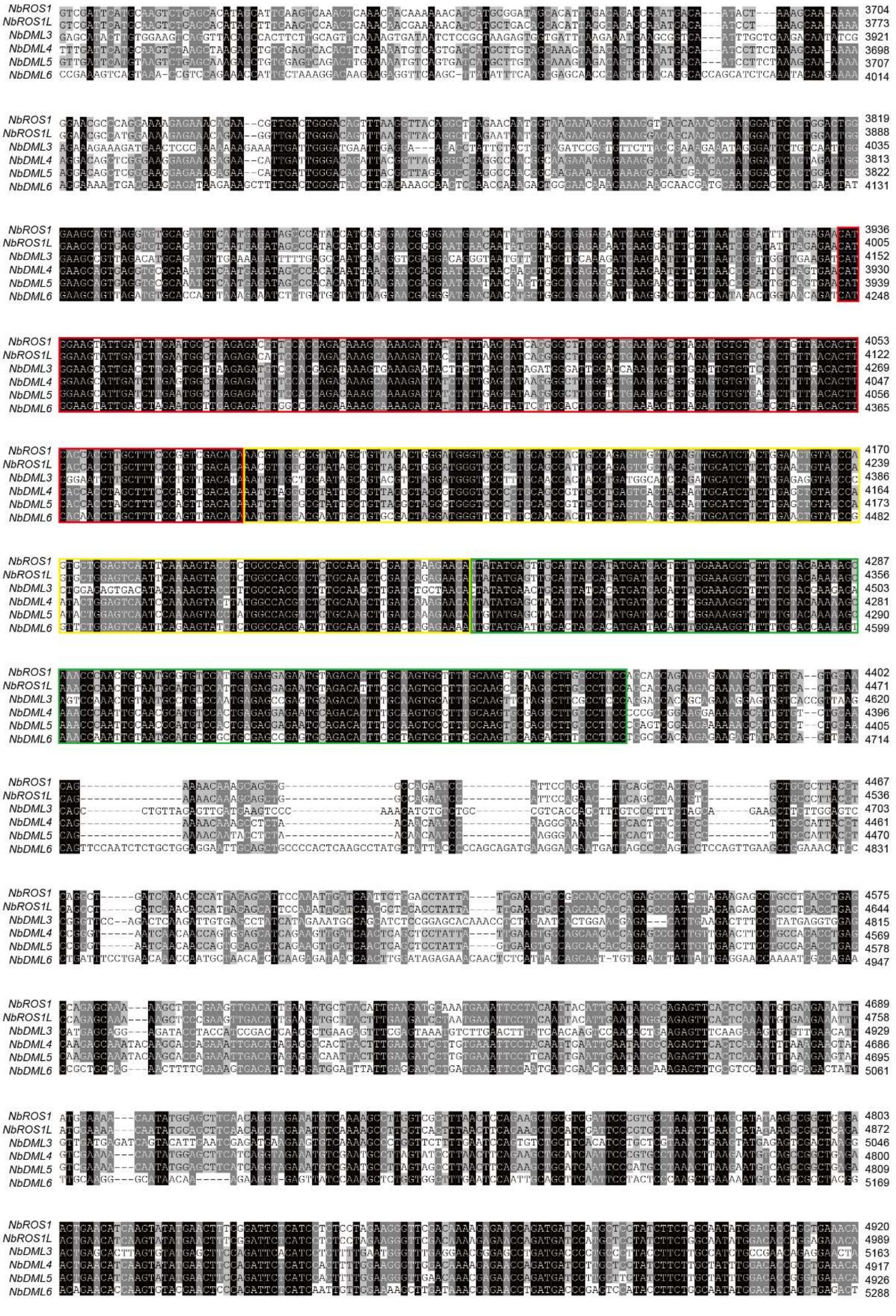
NbROS1
NbROS1L
NbDML3
NbDML4
NbDML5
NbDML6

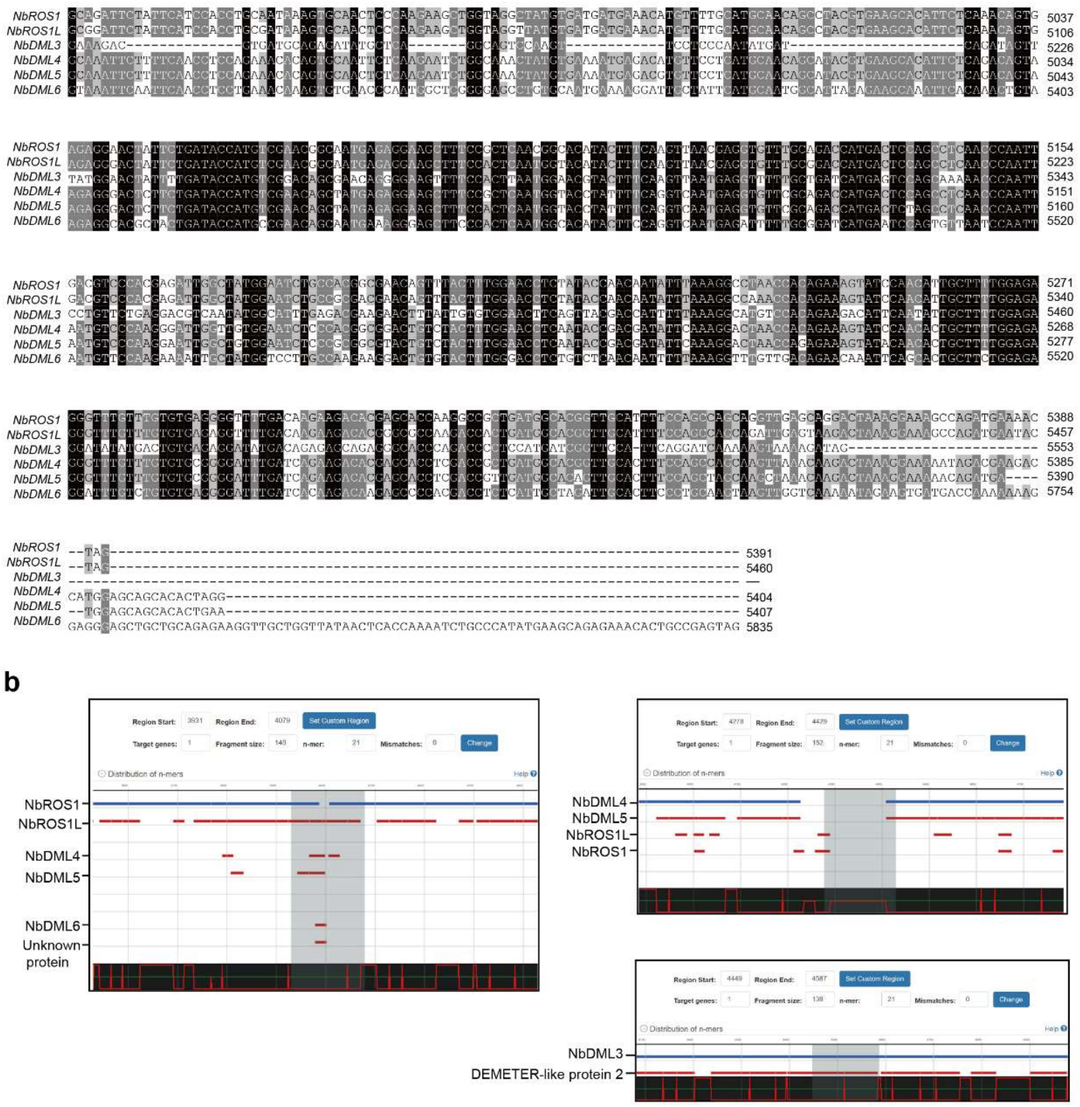

Supplementary Fig. 2 Alignment of *NbDMLs* and the putative siRNA hits. **a** Alignment of cDNA of *NbROS1*, *NbROS1L*, *NbDML3*, *NbDML4*, *NbDML5*, and *NbDML6* by Clustal X. The cDNA fragments of *NbROS1*, *NbDML3*, and *NbDML4*, which were marked with red, green and yellow rectangles respectively, were fused and cloned into the pTRV2 vector for *NbDML* silencing. **b** Putative siRNA hits as determined by the SGN VIGS tool. The cDNA fragments of *NbROS1*, *NbDML3*, and *NbDML4* used for *NbDML* silencing are shown in gray.

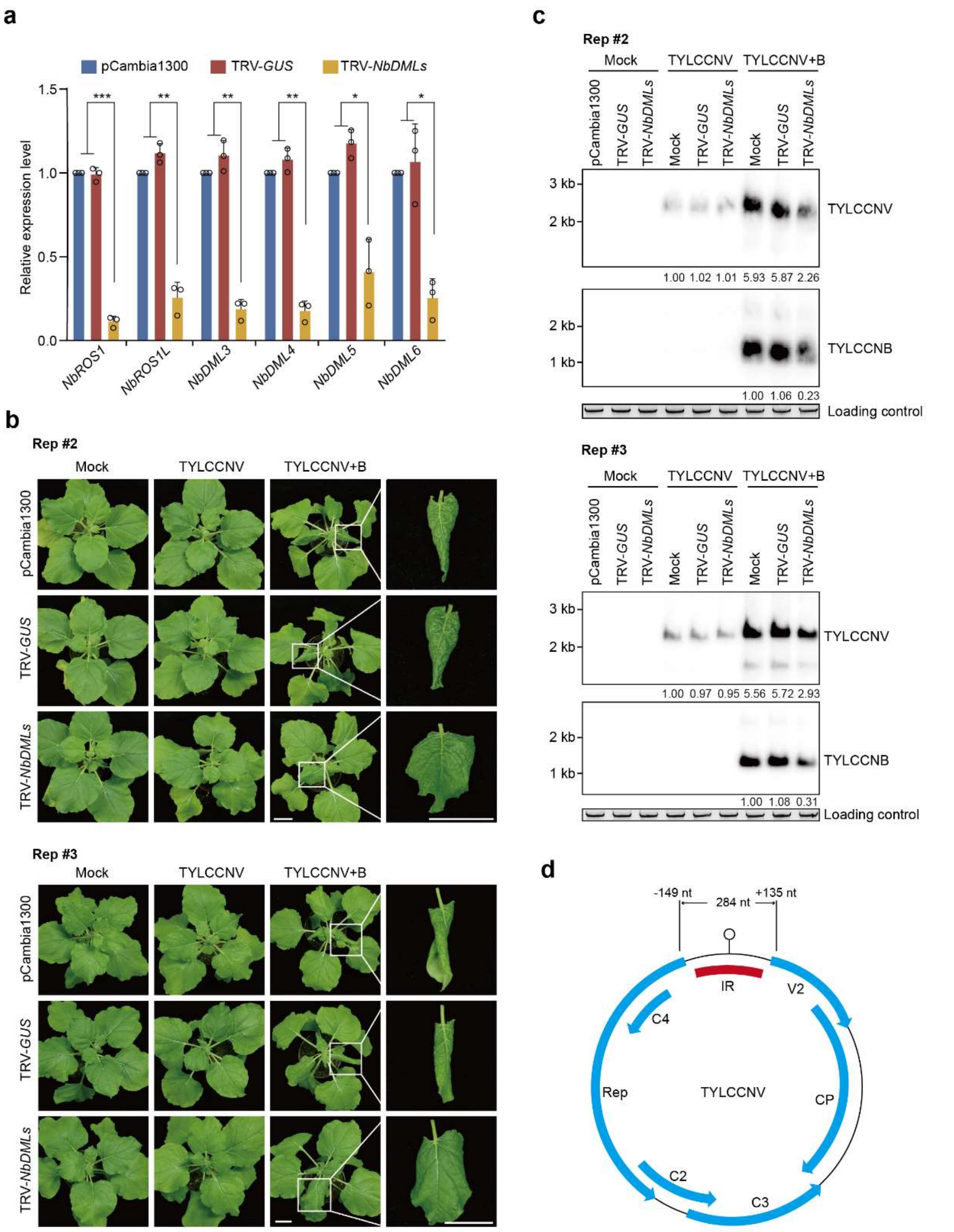

Supplementary Fig. 3 Active DNA demethylation promotes TYLCCNV+B virulence in *N. benthamiana*. **a** Transcript levels of *NbROS1*, *NbROS1L*, *NbDML3*, *NbDML4*, *NbDML5*, and *NbDML6* in *N. benthamiana* plants infiltrated with pCambia1300, TRV-*GUS* or TRV-*NbDMLs* as determined by RT-qPCR. Values are means ± SD (n = 3 independent experiments). Statistical significance was determined using two-tailed Student's *t*-test; **P* < 0.05, ***P* <

0.01 and ***$P < 0.001$. **b** Symptoms of pCambia1300-, TRV-*GUS*-, and TRV-*NbDMLs*-infiltrated *N. benthamiana* plants mock-inoculated, or inoculated with TYLCCNV or TYLCCNV+B at 10 dpi. Results from two additional replicates are shown. Photographs were taken at 10 day post inoculation (dpi). Scale bar, 2 cm. **c** Accumulation of TYLCCNV and TYLCCNB in the leaves of indicated plants at 10 dpi as determined by Southern blot. Results from two additional replicates are shown. Total DNA was stained with ethidium bromide (EB) as a loading control. **d** Genomic organization of TYLCCNV. The intergenic region (IR) region selected for bisulfite sequencing is indicated. Uncropped pictures, blots and gels for **b** and **c**, raw data and *P*-values for **a** are provided in the source data.

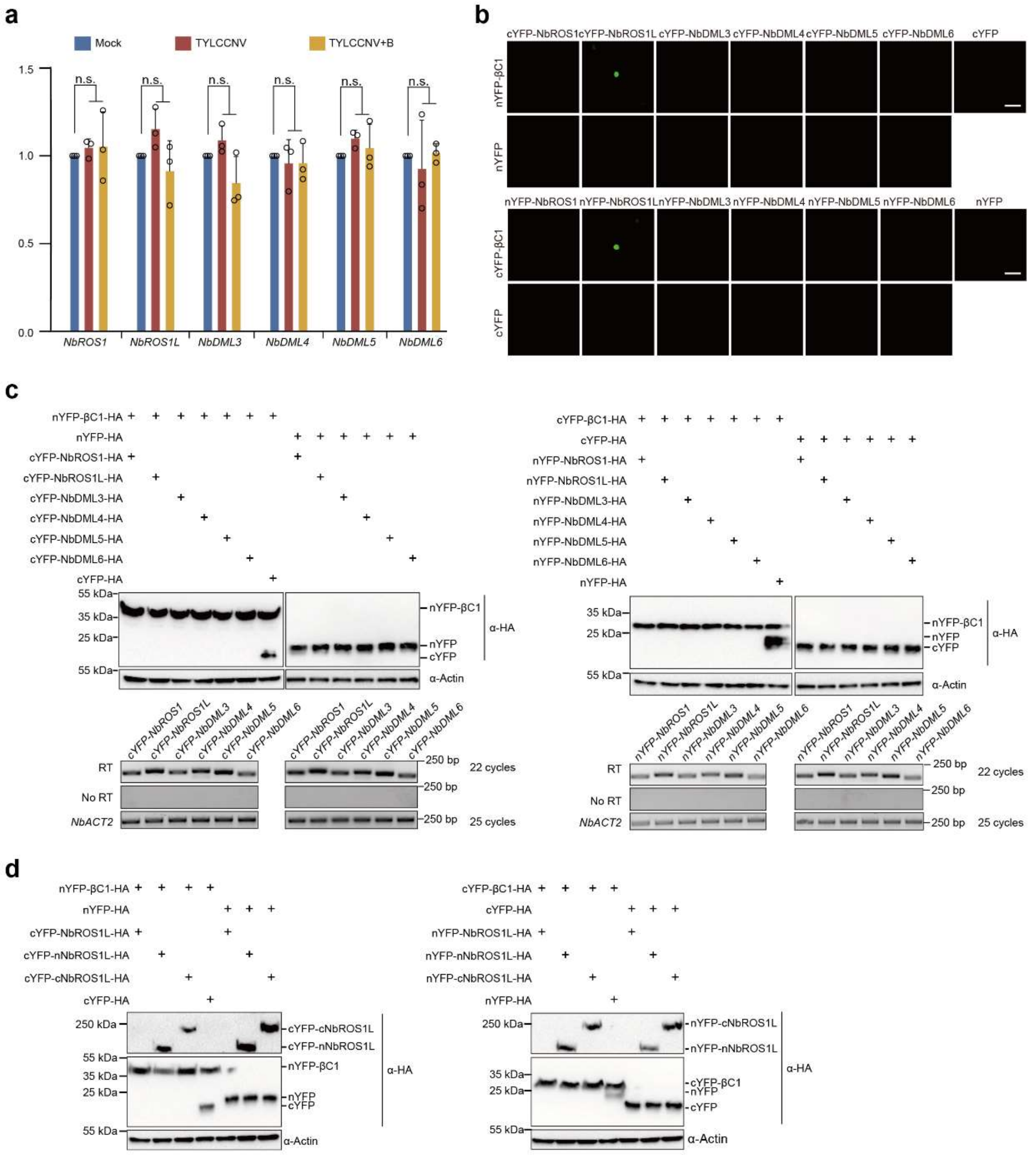

Supplementary Fig. 4 βC1 interacts with NbROS1L. a Transcript levels of *NbROS1*, *NbROS1L*, *NbDML3*, *NbDML4*, *NbDML5* and *NbDML6* in mock-, TYLCCNV- and TYLCCNV+B-inoculated *N. benthamiana* plants at 10 dpi. Values are means ± SD (n = 3 independent experiments). Statistical significance was determined using two-tailed Student's *t*-test; n.s.= not significant. **b** BiFC analysis of the interactions of βC1 with NbROS1, NbROS1L, NbDML3, NbDML4, NbDML5 and NbDML6. Scale bars, 50 μm. **c** Western blot and RT-PCR results showing the levels of proteins and transcripts involved in the BiFC experiments, related to Supplementary Fig. 4b. Actin and *NbACT2* served as controls. **d** Western blot results showing the levels of proteins

involved in the BiFC experiments, related to Fig. 2b. Actin served as a control. Images in **b**-**d** are representative of three independent experiments. Raw data for **b**, uncropped blots and gels for **c** and **d**, raw data and *P*-values for **a** are provided in the source data.

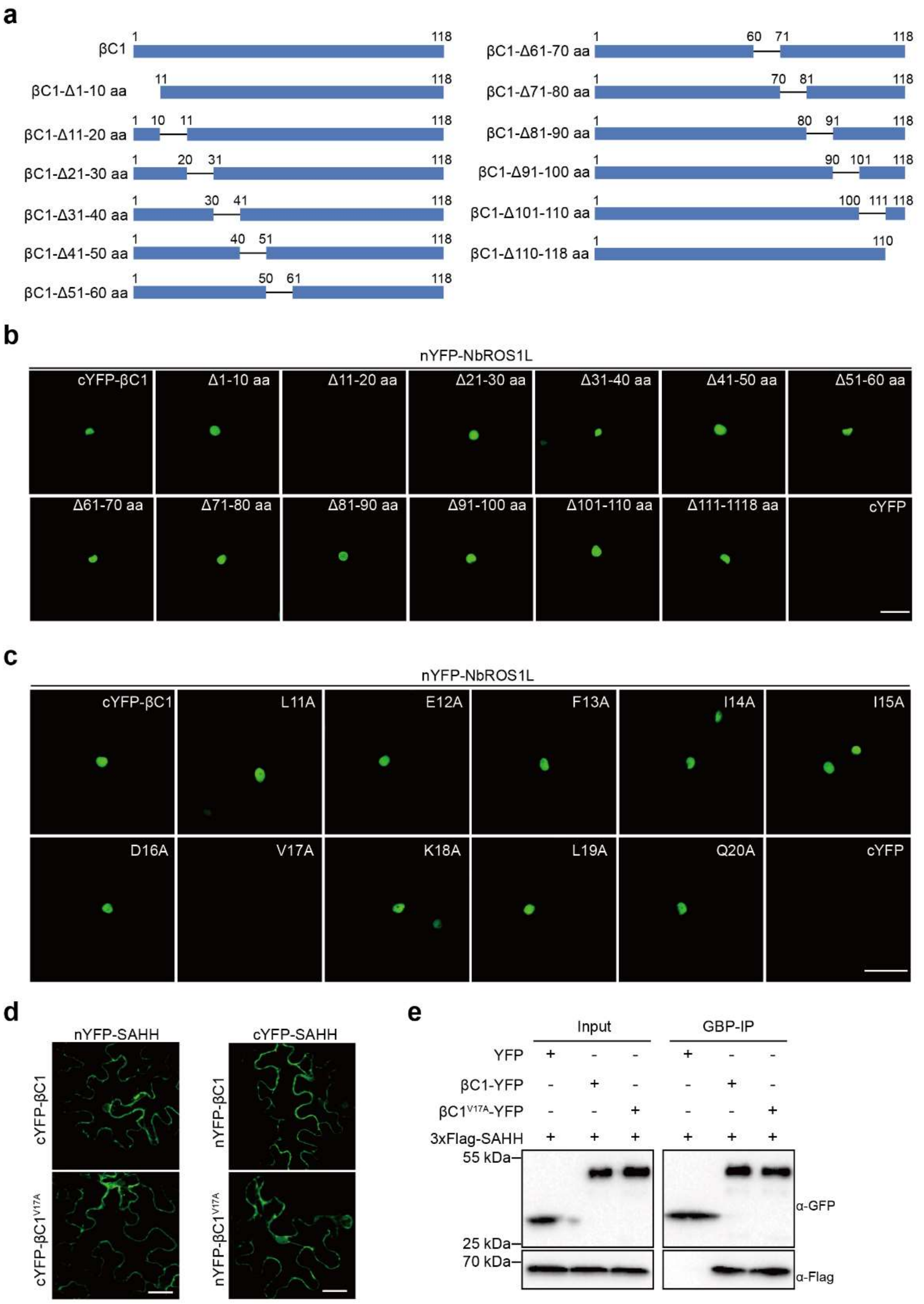

Supplementary Fig. 5 The V17A mutation abolishes the interaction of βC1 with NbROS1L but does not affect its interaction with SAHH. a Schematic representation of different truncated forms of βC1, related to Supplementary Fig. 5b. **b** BiFC analysis of the interactions between different truncated forms of βC1 and NbROS1L. Scale bar, 50 μm. **c** BiFC analysis of the interactions between different point mutants of βC1 and NbROS1L. Scale bar, 50 μm. **d**

BiFC analysis of the interaction of βC1 or $\beta C1^{V17A}$ with SAHH. Scale bars, 50 μm. **e** Co-immunoprecipitation analysis of the interactions of βC1 or $\beta C1^{V17A}$ with SAHH. YFP was used as a negative control. Images in **b**-**e** are representative of three independent experiments. Raw data for **b**-**d** and uncropped blots for **e** are provided in the source data.

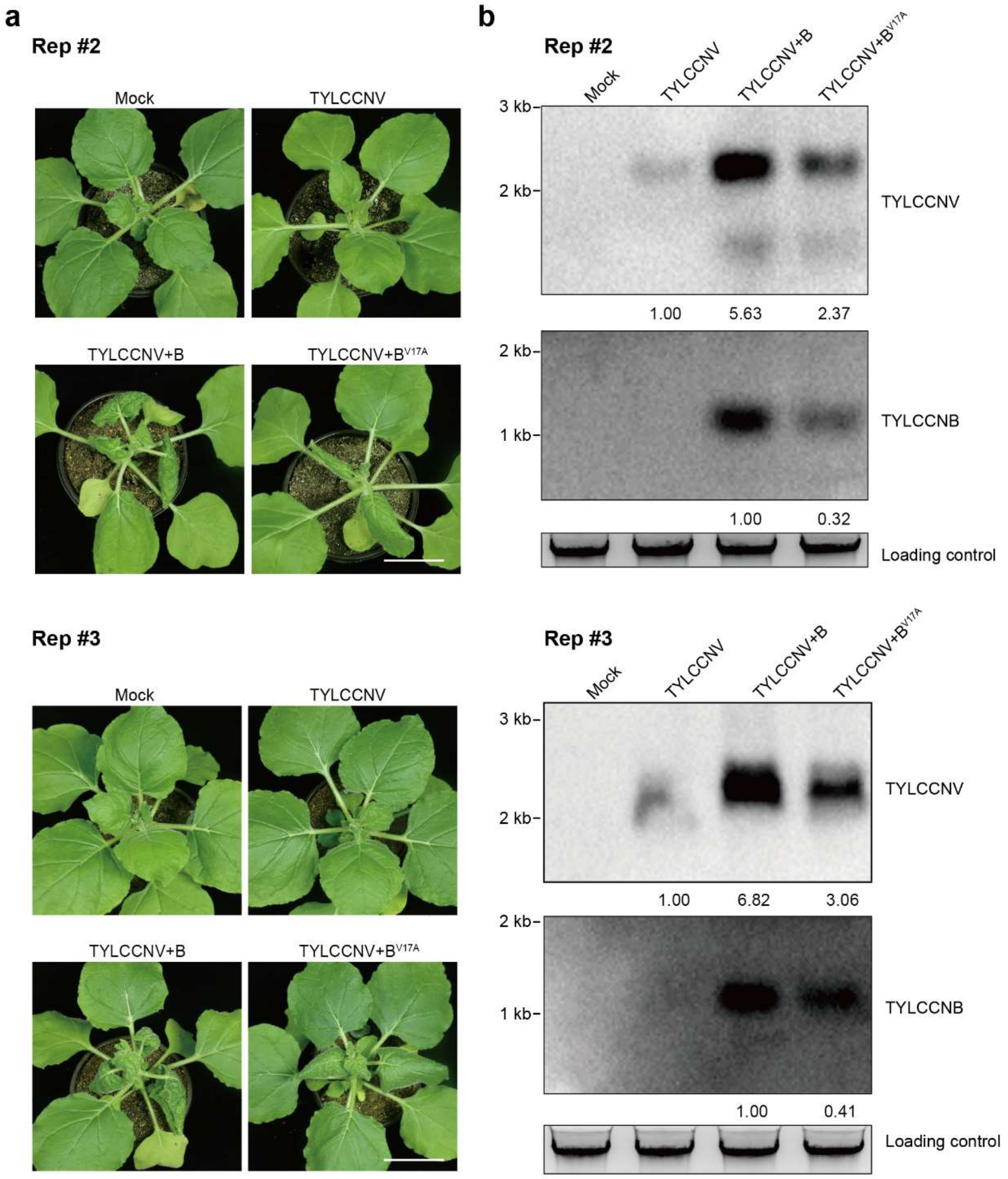

Supplementary Fig. 6 The βC1-NbROS1L interaction is important for TYLCCNV+B virulence. a Symptoms of *N. benthamiana* plants mock-inoculated or inoculated with TYLCCNV, TYLCCNV+B or TYLCCNV+B^{V17A} at 10 dpi. Results from two additional replicates are shown. Scale bar, 2 cm. **b** Accumulation of TYLCCNV and TYLCCNB in the leaves of indicated plants at 10 dpi as determined by Southern blot. Results from two additional replicates are shown. Total DNA was stained with EB as a loading control. Uncropped pictures, blots and gels for **a** and **b** are provided in the source data.

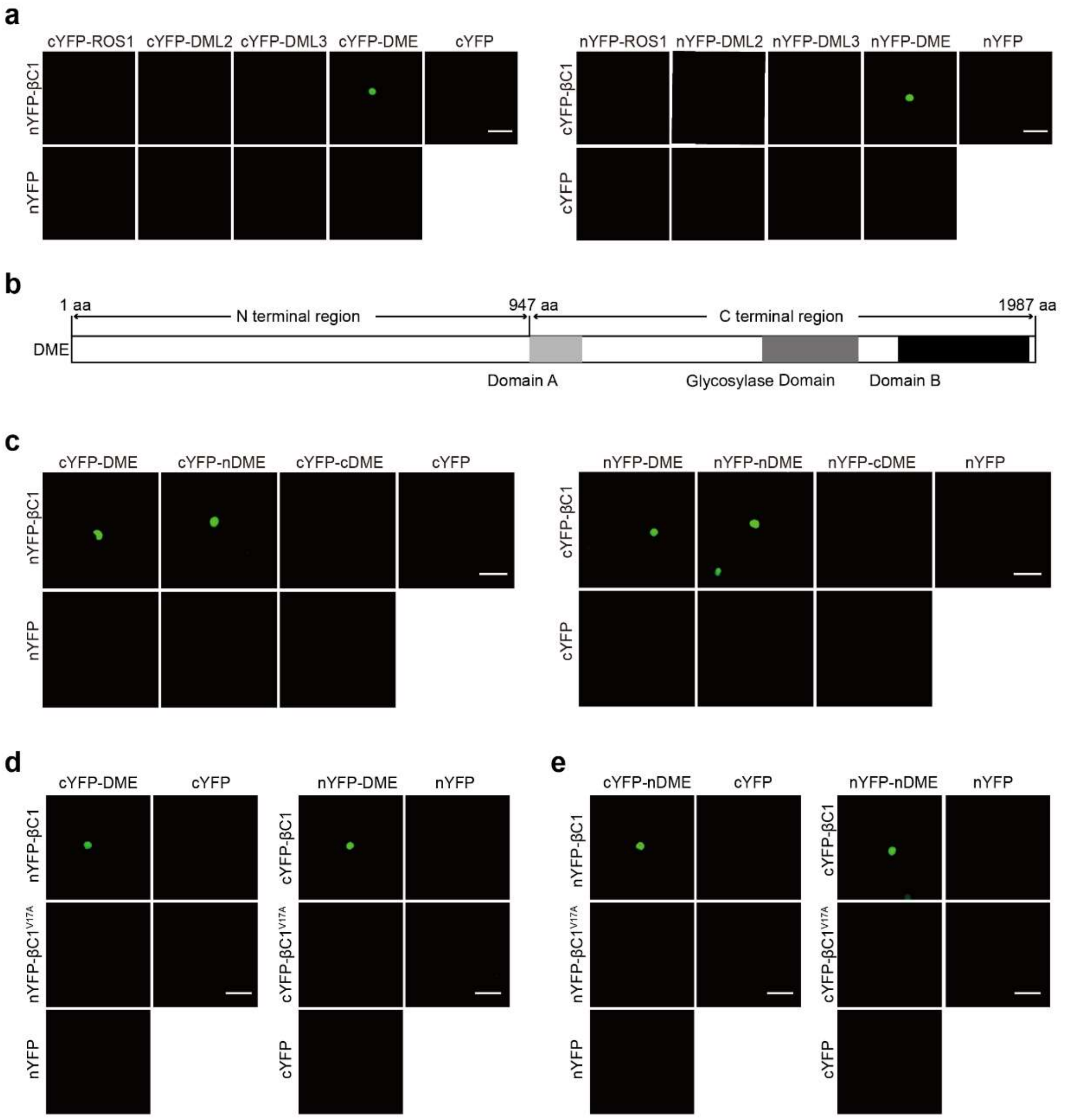

Supplementary Fig. 7 βC1 interacts with DME. **a** BiFC analysis of the interactions of βC1 with ROS1, DML2, DML3 and DME. Scale bars, 50 μm. **b** Schematic representation of DME protein structure. **c** BiFC analysis of the interactions of βC1 with the N-terminal region of DME (nDME) and the C-terminal region of DME (cDME). Scale bars, 50 μm. **d** BiFC analysis of the interactions of βC1 or βC1^{V17A} with DME. Scale bars, 50 μm. **e** BiFC analysis of the interactions of βC1 or βC1^{V17A} with nDME. Scale bars, 50 μm. Images in **a** and **c**-**e** are representative of three independent experiments. Raw data for **a** and **c**-**e** are provided in the source data.

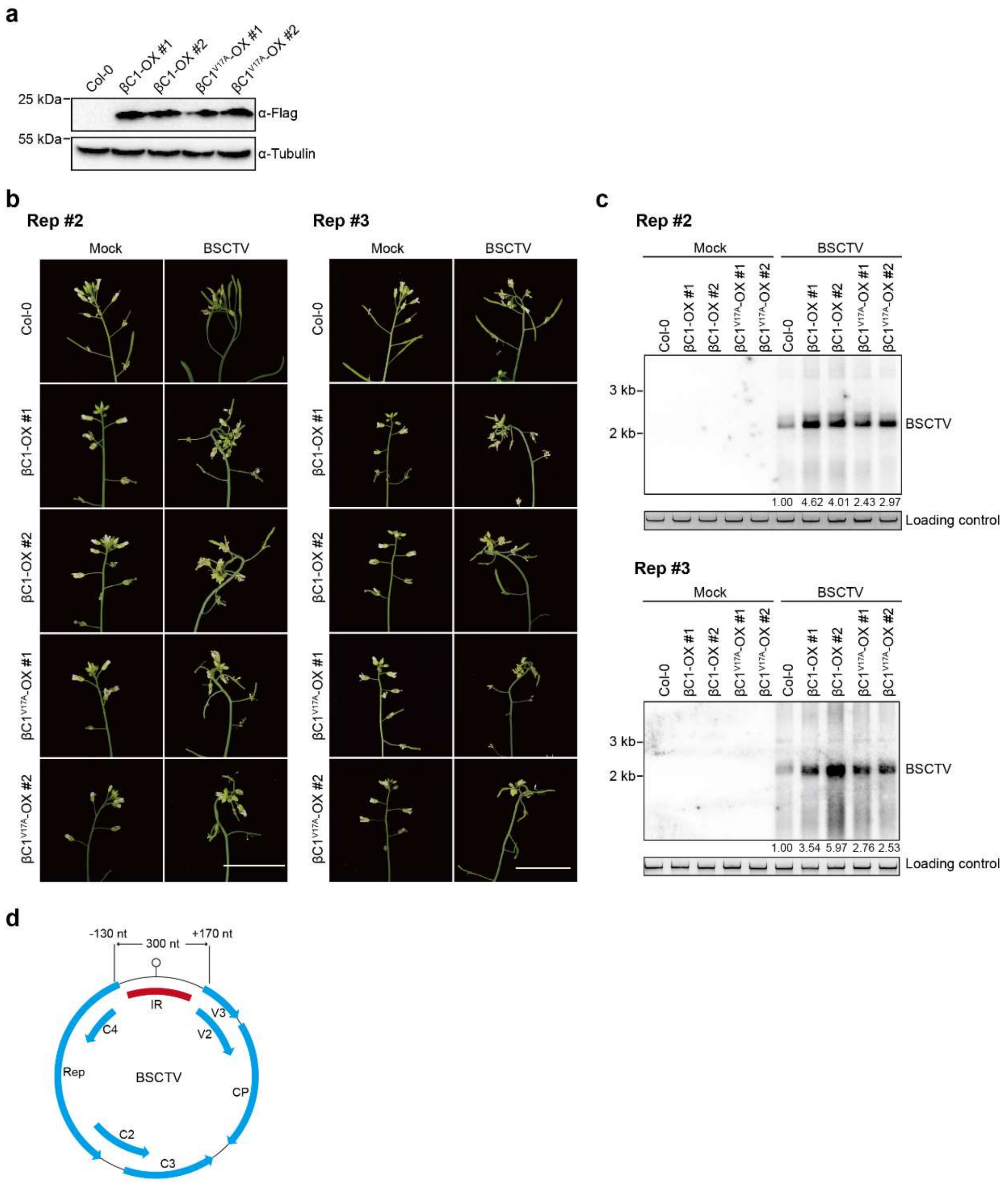

Supplementary Fig. 8 The βC1-DME interaction promotes BSCTV virulence in *Arabidopsis*. **a** Protein level of βC1 in the indicated plants as determined by western blot. Tubulin served as a loading control. Images are representative of three independent experiments. **b** Symptoms of Col-0, βC1-expressing lines, and βC1^{V17A}-expressing lines inoculated with BSCTV at 10 dpi. Results from two additional replicates are shown. Scale bars, 2 cm. **c** Accumulation of BSCTV in the indicated plants at 10 dpi as determined by Southern blot. Results from two additional replicates are shown. Total DNA was

stained with EB as a loading control. **d** Genomic organization of BSCTV. The IR region selected for bisulfite sequencing is indicated. Uncropped pictures, blots and gels for **a**-**c** are provided in the source data.

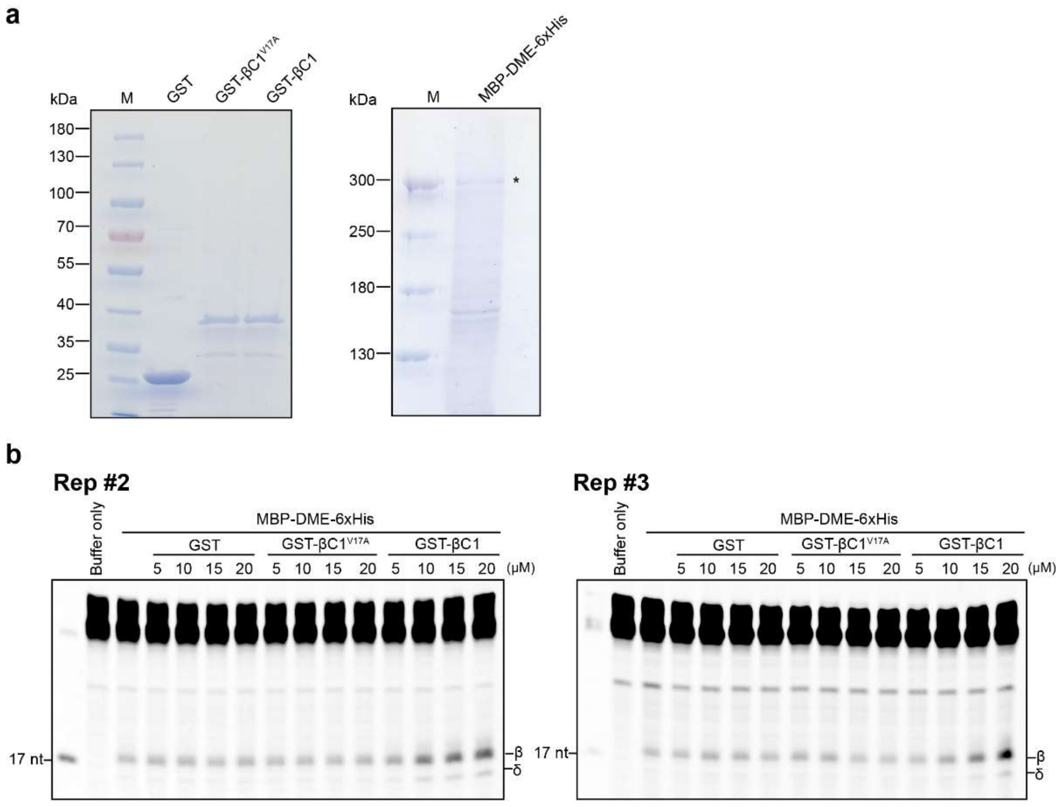

Supplementary Fig. 9 βC1 promotes DME activity *in vitro*. a Coomassie blue staining of GST, GST-βC1, GST-βC1^{V17A} and MBP-DME-6xHis. Images are representative of three independent experiments. **b** *In vitro* 5mC excision activity of DME proteins. Results from two additional replicates are shown. Double-stranded oligonucleotide substrate (one strand methylated) was incubated with DME and varying amounts of GST, GST-βC1 or GST-βC1^{V17A}. 17-nucleotide size marker and β- and δ-elimination products are indicated. Uncropped blots and gels for **a** and **b** are provided in the source data.

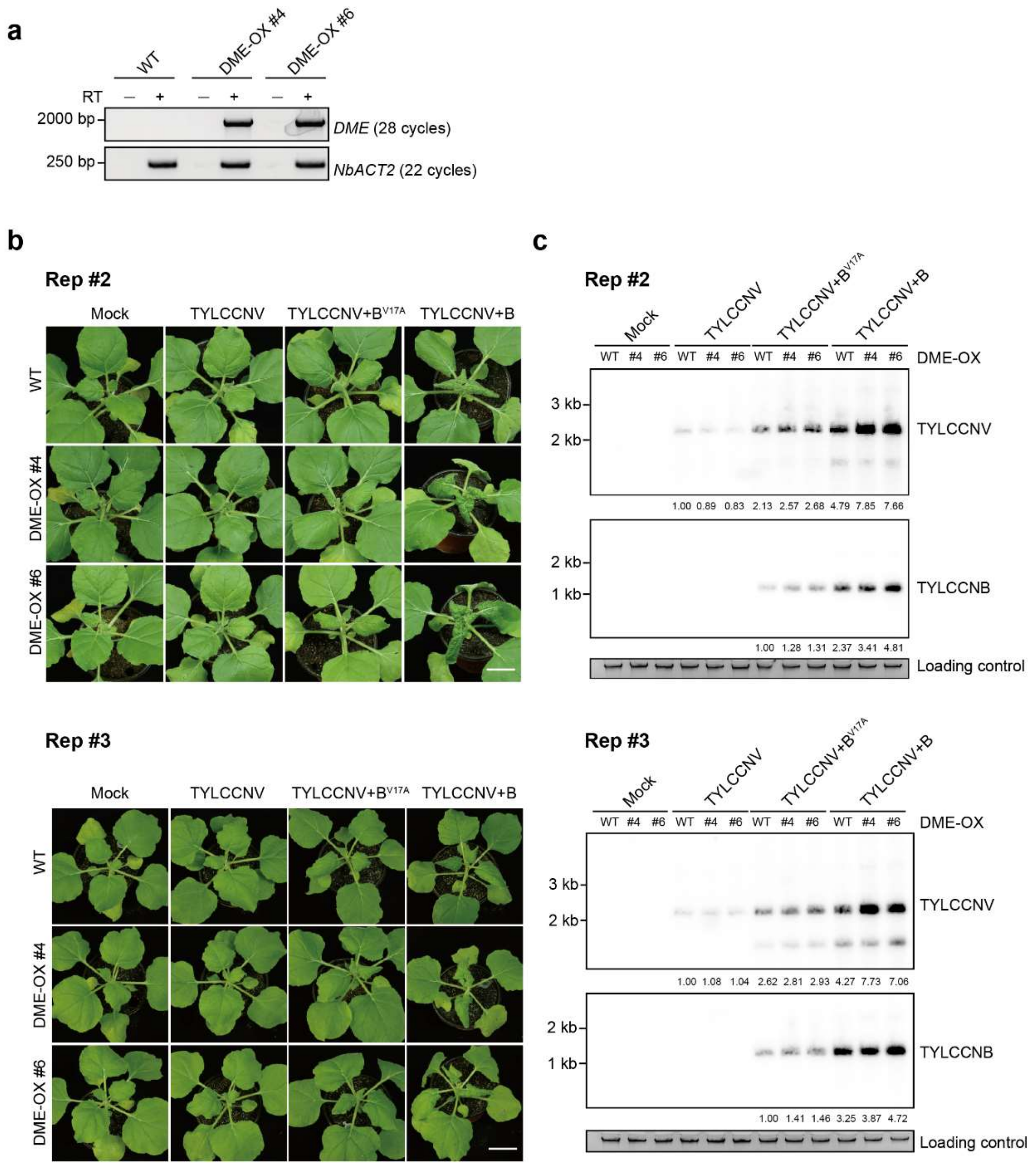

Supplementary Fig. 10 DME overexpression promotes TYLCCNV+B virulence. a Transcript levels of *DME* in the indicated plants as determined by RT-PCR. *NbACT2* served as a control. Images are representative of three independent experiments. **b** Symptoms of wild-type *N. benthamiana* plants and DME-overexpressing lines mock-inoculated or inoculated with TYLCCNV, TYLCCNV+B or TYLCCNV+B^{V17A} at 10 dpi. Results from two additional replicates are shown. Scale bar, 2 cm. **c** Accumulation of TYLCCNV and TYLCCNB in the leaves of indicated plants at 10 dpi as determined by Southern blot. Results from two additional replicates are shown. Total DNA was stained

with EB as a loading control. Uncropped pictures, blots and gels for **a**-**c** are provided in the source data.

Supplementary Table 1

Primers used for generating TRV-based VIGS vector

Primer	Sequence (5'-3')
TRV-NbDMLs-XbaI-F1	AAGGTTACCGAATTCTCTAGAGAACATGGAAGTATTGATCTTG
NbDMLs-overlap-R1	ACCGCAATACGCCCTACATTTGTGTCGACCGGGAAAGCAA
NbDMLs-overlap-F2	TTGCTTTCCCGGTCGACACAAATGTAGGGCGTATTGCGGT
NbDMLs-overlap-R2	TGTGATAATGCAGTTCATATAATGTTCTTTGATCAAGCTTG
NbDMLs-overlap-F3	CAAGCTTGATCAAAGAACATTATATGAACTGCATTATCACA
TRV-NbDMLs-XhoI-R3	GGGACATGCCCGGGCCTCGAGGGGAGGCGAAGCCTAGAACTTG

Primers used for RT-PCR and qPCR

Primer	Sequence (5'-3')
NbROS1-qPCR-F	CCTTTCTAGCTCGGCATTCA
NbROS1-qPCR-R	GCCACTCACTTCAGGTTCTT
NbROS1L-qPCR-F	CCTTGCCAGTTCGCAGAATG
NbROS1L-qPCR-R	TGATCTGGTCTGGTGTTATTGG
NbDML3-qPCR-F	AGAGCCTGTCAGCATCATATTC
NbDML3-qPCR-R	CGTCGTTCCATGGTCATTC
NbDML4-qPCR-F	GGACTCCGTAGTTGGAGTATTTC
NbDML4-qPCR-R	CTGTTCTCCCTTCATGCTTCT
NbDML5-qPCR-F	GCAGAAGTCAATGCCAAACTAC
NbDML5-qPCR-R	CTATCCTGGCCCTTTCTGTAAC
NbDML6-qPCR-F	CTCTCAAGCTCCGCTTTCAT
NbDML6-qPCR-R	CTCCACTACTGTGTTCCCATTC
NbACT2-qPCR-F	CAATCCAGACACTGTACTTTCTCTC
NbACT2-qPCR-R	AAGCTGCAGGTATCCATGAGACTA
DME-PCR-F	ATGAATTCGAGGGCTGATCCG
DME-PCR-R	TCCTGGAAACAGATTCCACATG
NbACT2-PCR-F	CAATCCAGACACTGTACTTTCTCTC
NbACT2-PCR-R	AAGCTGCAGGTATCCATGAGACTA

Primers used for generating Southern blot probes

Primer	Sequence (5'-3')
TYLCCNV-Southern-F	ATGTGGGATCCTCTGCTCAACGAGTTTC
TYLCCNV-Southern-R	CATCCTCAGACCTTGCGTTTCTTAAGAG
TYLCCNB-Southern-F	GAAACCACTACGCTACGCAGC
TYLCCNB-Southern-R	TACCCTCCCAGGGGTACACAC
BSCTV-Southern-F	ATGGATTTGGGCGGGAAACTTC
BSCTV-Southern-R	GCCAAACAAAGTGGCATACAT

Primers used for bisulfite sequencing

Primer	Sequence (5'-3')
TYLCCNV-Bisulfite-F1	TCGTCGGCAGCGTGAGATGTGTATAAGAGACAGTCTCTCCGGYATT GAYTGGTYAATTGGTGT
TYLCCNV-Bisulfite-R1	GTCTCGTGGGCTCGGAGATGTGTATAAGAGACAGTCCTCTACTCCC ACATRCTTAACRTRAATAC
TYLCCNV-Bisulfite-F2	TCGTCGGCAGCGTGAGATGTGTATAAGAGACAGCGTCTAATGYATT GAYTGGTYAATTGGTGT
TYLCCNV-Bisulfite-R2	GTCTCGTGGGCTCGGAGATGTGTATAAGAGACAGTGCCTCTTTCCC ACATRCTTAACRTRAATAC
TYLCCNV-Bisulfite-F3	TCGTCGGCAGCGTGAGATGTGTATAAGAGACAGCTAAGCCTGYATT GAYTGGTYAATTGGTGT
TYLCCNV-Bisulfite-R3	GTCTCGTGGGCTCGGAGATGTGTATAAGAGACAGCAGCCTCGTCCC ACATRCTTAACRTRAATAC
BSCTV-Bisulfite-F	TCGTCGGCAGCGTGAGATGTGTATAAGAGACAGGGTATGTAAGGAA AAAATTTTTGG
BSCTV-Bisulfite-R	GTCTCGTGGGCTCGGAGATGTGTATAAGAGACAGTAACTTTATTTCA AACARTATRTC

Primers used for BiFC assay

Primer	Sequence (5'-3')
βC1-BiFC-PacI-F	ATTTACGAACGATAGTTAATTAACATGACTATCAAATACAACAAC
βC1-BiFC-SpeI-R	ACTGCCACCTCCTCCACTAGTTACATCTGAATTTGTAAATACATC
βC1-Δ1-10-BiFC-PacI-F	ATTTACGAACGATAGTTAATTAACATGTTGGAGTTCATCATTGATGT
βC1-Δ110-118-BiFC-SpeI-R	ACTGCCACCTCCTCCACTAGTATACTCATCCCCTACATCTAT
NbROS1-BiFC-PacI-F	ATTTACGAACGATAGTTAATTAACATGGAGGGTAGGCAAAGAAG
NbROS1-BiFC-SpeI-R	ACTGCCACCTCCTCCACTAGTGTTTTCATCTGGCTTTCCTTT
NbROS1L-BiFC-PacI-F	ATTTACGAACGATAGTTAATTAACATGGAGGGAAGTGAAAGAAGC
NbROSL1-BiFC-SpeI-R	ACTGCCACCTCCTCCACTAGTGTATTCATCTGGCTTTCCTT
cNbROSL1-BiFC-PacI-F	ATTTACGAACGATAGTTAATTAACATGAGAAGACGAAAACCGCGG
nNbROSL1-BiFC-SpeI-R	ACTGCCACCTCCTCCACTAGTAACGAATGATCCTGCAGAAGG
NbDML3-BiFC-PacI-F	ATTTACGAACGATAGTTAATTAACATGGAAGTGGGCAATGTAGG
NbDML3-BiFC-SpeI-R	ACTGCCACCTCCTCCACTAGTTCTTTTACTTTTTGATCCTG
NbDML4-BiFC-PacI-F	ATTTACGAACGATAGTTAATTAACATGGATGTAGGCCAAAGCAG
NbDML4-BiFC-SpeI-R	ACTGCCACCTCCTCCACTAGTGTGTGCTGCTCCATGGTCTTC
NbDML5-BiFC-PacI-F	ATTTACGAACGATAGTTAATTAACATGGATGTAGGCCAAGGCAGC
NbDML5-BiFC-SpeI-R	ACTGCCACCTCCTCCACTAGTGTGTGCTGCTCCATCATCTGT
NbDML6-BiFC-PacI-F	ATTTACGAACGATAGTTAATTAACATGAATCTGGGGAGATTTTC
NbDML6-BiFC-SpeI-R	ACTGCCACCTCCTCCACTAGTCTCGGCAGTGTTTCTCTGCTTC
AtROS1-BiFC-PacI-F	ATTTACGAACGATAGTTAATTAACATGGAGAAACAGAGGAGAGAAG
AtROS1-BiFC-SpeI-R	ACTGCCACCTCCTCCACTAGTGGCGAGGTTAGCTTGTTGTCC
AtDML2-BiFC-PacI-F	ATTTACGAACGATAGTTAATTAACATGGAAGTGGAAGGTGAAGTG
AtDML2-BiFC-SpeI-R	ACTGCCACCTCCTCCACTAGTTTCCTCTGTCTTCTCTTTAG

AtDML3-BiFC-Pacl-F	ATTTACGAACGATAGTTAATTAACATGTTGACAGATGGTTCACAAC
AtDML3-BiFC-Spel-R	ACTGCCACCTCCTCCACTAGTTATATCATCATCACTCATAAAC
DME-BiFC-Pacl-F	ATTTACGAACGATAGTTAATTAACATGAATTCGAGGGCTGATCCG
DME-BiFC-Spel-R	ACTGCCACCTCCTCCACTAGTGGTTTTGTTGTTCTTCAATTTG
cDME-BiFC-Pacl-F	ATTTACGAACGATAGTTAATTAACATGAAGAAGCGAAAACCAAGACCC
nDME-BiFC-Spel-R	ACTGCCACCTCCTCCACTAGTGCTCTCATAGGGAACAAGTGC
SAHH-BiFC-Pacl-F	ATTTACGAACGATAGTTAATTAACATGGCGTTGCTCGTCGAGAAG
SAHH-BiFC-Spel-R	ACTGCCACCTCCTCCACTAGTGTACCTGTAGTGAGGAGGCTTG

Primers used for generating truncated and mutant forms of βC1

Primer	Sequence (5'-3')
pLB-βC1-Δ1-10-F	TTGGAGTTCATCATTGATGT
pLB-βC1-Δ1-10-R	ATCTTGCTGAAAAACTCGAG
pLB-βC1-Δ11-20-F	GAAGACACTTCAATCCTTGT
pLB-βC1-Δ11-20-R	ACCCTTCATGTTGTTGTATTTG
pLB-βC1-Δ21-30-F	CTAGCATCAACAAAGTCACC
pLB-βC1-Δ21-30-R	CTGTAACTTGACATCAATGAT
pLB-βC1-Δ31-40-F	AAGAAGAAATTCATGATCCC
pLB-βC1-Δ31-40-R	TTCAATCCTTACAAGGATTG
pLB-βC1-Δ41-50-F	CATGGGATTATCCCACCATTC
pLB-βC1-Δ41-50-R	GGCTAATGCTGGTGACTTTG
pLB-βC1-Δ51-60-F	ATTTTAGAAGAAGGCATCAAA
pLB-βC1-Δ51-60-R	GTGTCTGTATGGGATCATGAA
pLB-βC1-Δ61-70-F	AAAATAATGTATAAGGAGTCT
pLB-βC1-Δ61-70-R	GTTGAAGTCGAATGGTGGGA
pLB-βC1-Δ71-80-F	GAATTTAGGCAAGAAGATATG
pLB-βC1-Δ71-80-R	GAGGCAGTTTTTGATGCCTTC
pLB-βC1-Δ81-90-F	ATAGACATAATCATGATGGAAG
pLB-βC1-Δ81-90-R	CCCAATTGTAGACTCCTTAT
pLB-βC1-Δ91-100-F	GTAGAAGATATAGATGTAGGG
pLB-βC1-Δ91-100-R	AACTTGTACCATATCTTCTTG
pLB-βC1-Δ101-110-F	GATGTATTTACAAATTCAGATG
pLB-βC1-Δ101-110-R	TGGGGCTTCTTCCATCATGA
pLB-βC1-Δ111-118-F	ATCTTTTAGAAGATCTCCTAC
pLB-βC1-Δ111-118-R	ATACTCATCCCCTACATCTAT
pLB-βC1-L11A-F	GCGGAGTTCATCATTGATGTCAAGT
pLB-βC1-L11A-R	ACCCTTCATGTTGTTGTATTT
pLB-βC1-E12A-F	GCGTTCATCATTGATGTCAAG
pLB-βC1-E12A-R	CAAACCCTTCATGTTGTTGTAT
pLB-βC1-F13A-F	GCCATCATTGATGTCAAGTTACAG
pLB-βC1-F13A-R	CTCCAAACCCTTCATGTTGTT
pLB-βC1-I14A-F	GCCATTGATGTCAAGTTACAGGAAG
pLB-βC1-I14A-R	GAACTCCAAACCCTTCATGTT

pLB-βC1-I15A-F	GCTGATGTCAAGTTACAGGAAGAC
pLB-βC1-I15A-R	GATGAACTCCAAACCCTTCATG
pLB-βC1-D16A-F	GCTGTCAAGTTACAGGAAGAC
pLB-βC1-D16A-R	AATGATGAACTCCAAACCCTTC
pLB-βC1-V17A-F	GCCAAGTTACAGGAAGACACTTCA
pLB-βC1-V17A-R	ATCAATGATGAACTCCAAACC
pLB-βC1-K18A-F	GCGTTACAGGAAGACACTTCAATCC
pLB-βC1-K18A-R	GACATCAATGATGAACTCCAAAC
pLB-βC1-L19A-F	GCACAGGAAGACACTTCAATCCTT
pLB-βC1-L19A-R	CTTGACATCAATGATGAACTC
pLB-βC1-Q20A-F	GCGGAAGACACTTCAATCCTTGT
pLB-βC1-Q20A-R	TAACTTGACATCAATGATGA

Primers used for co-IP experiments

Primer	Sequence (5'-3')
5myc-nNbROS1L-SalI-F	AAGCTTATCGATACCGTCGACATGGAGGGAAGTGAAAGAAGC
5myc-nNbROS1L-KpnI-R	TTTGCGGAGTACCCGGGTACCCTAAACGAATGATCCTGCAGA
5myc-cNbROS1L-SalI-F	AAGCTTATCGATACCGTCGACATGAGAAGACGAAAACCGCGGCC
5myc-cNbROS1L-KpnI-R	TTTGCGGAGTACCCGGGTACCCTAGTATTCATCTGGCTTTCCTT
5myc-DME-EcoRI-F	TCCCCCGGGCTGCAGGAATTCATGAATTCGAGGGCTGATCCG
5myc-DME-SalI-R	GGGCCCCCCCTCGAGGTCGACTTAGGTTTTGTTGTTCTTCAAT
5myc-nDME-SalI-R	GGGCCCCCCCTCGAGGTCGACTTAGCTCTCATAGGGAACAAGTGC
βC1-YFP-BamHI-F	GCGCTGCAGGGAGGAGGATCCATGACTATCAAATACAACAAC
βC1-YFP-SalI-R	GACCGGTGCACTAGTGTCGACTACATCTGAATTTGTAAATACATC
3Flag-SAHH-SmaI-F	GATGACGACGATAAGCCCGGGATGGCGTTGCTCGTCGAGAAG
3Flag-SAHH-SalI-R	AGGGCATGCCTGCAGGTCGACTCAGTACCTGTAGTGAGGAGGC

Primers used for generating infectious clone of $TYLCCNB^{V17A}$

Primer	Sequence (5'-3')
pLB-TYLCCNB-F	GGTACCACTACGCTACGCAGCAGCC
pLB-TYLCCNB-R	GAATTCTACCCTCCCAGGGGTACACAC
pBinPLUS-TYLCCNB-F	CTTGCATGCCTGCAGGTCGACGAAACCACTACGCTACGCAG
pBinPLUS-TYLCCNB-R	TAAGAATTCGAGCTCGGTACCTACCCTCCCAGGGGTACACA

Primers used for the expression of recombinant proteins in *E. coli*

Primer	Sequence (5'-3')
GST-βC1-BamHI-F	GATCTGGTTCCGCGTGGATCCATGACTATCAAATACAACAAC
GST-βC1-SalI-R	GATGCGGCCGCTCGAGTCGACTCATACATCTGAATTTGTAA
pET11-Nde1-DME-F	CTCTACTTCCAATCCCATATGATGAATTCGAGGGCTGATCCG
pET11-XhoI-DME-R	ATGGTGATGGTGATGCTCGAGGGTTTTGTTGTTCTTCAATTTG

Primers used for generating *βC1* and *βC1*^{V17A} transgenic plants

Primer	Sequence (5'-3')
3Flag-βC1-SmaI-F	GATGACGACGATAAGCCCGGGATGACTATCAAATACAACAAC
3Flag-βC1-SalI-R	AGGGCATGCCTGCAGGTCGACTCATACATCTGAATTTGTAAATAC

Substrates for DME activity assay

Oligonucleotide	Sequence (5'-3')
MEA-1.6-F	CTATACCTCCTCAACTCCGGTCACCGTCTCCGGCG
MEA-1.6(5mC)-F	CTATACCTCCTCAACTC(5mC)CGGTCACCGTCTCCGGCG
MEA-1.6-R	CGCCGGAGACGGTGACCGGAGTTGAGGAGGTATAG
